# *Dorema Aucheri* Extract Modulates Unfolded Protein Response Pathways in Streptozotocin‐Induced Diabetic Rats: A Protective Role Against Hepatic Damage

**DOI:** 10.1155/ije/5570446

**Published:** 2026-09-16

**Authors:** Alireza Raeisi, Farhad Koohpeyma, Roozbeh Kiani, Somayeh Igder, Amir Maleksabet, Nima Rahimikashkooli, Narges Rezaei, Mesbah Shams, Sanaz Dastghaib

**Affiliations:** ^1^ Endocrinology and Metabolism Research Center, Shiraz University of Medical Sciences, Shiraz, Iran, sums.ac.ir; ^2^ Student Research Committee, Shiraz University of Medical Sciences, Shiraz, Iran, sums.ac.ir; ^3^ Autophagy Research Center, Shiraz University of Medical Sciences, Shiraz, Iran, sums.ac.ir; ^4^ Hyperlipidemia Research Center, Ahvaz Jundishapur University of Medical Sciences, Ahvaz, Iran, ajums.ac.ir; ^5^ Department of Clinical Biochemistry, Faculty of Medicine, Ahvaz Jundishapur University of Medical Sciences, Ahvaz, Iran, ajums.ac.ir; ^6^ Department of Medical Biotechnology, School of Advanced Technologies in Medicine, Mazandaran University of Medical Sciences, Sari, Iran, mazums.ac.ir; ^7^ Internal Medicine Department, School of Medicine, Shiraz University of Medical Sciences, Shiraz, Iran, sums.ac.ir; ^8^ Hematology Research Center, Shiraz University of Medical Sciences, Shiraz, Iran, sums.ac.ir

**Keywords:** antioxidant, diabetes, *Dorema aucheri*, gene expression, liver enzymes, oxidative stress

## Abstract

**Purpose:**

Endoplasmic reticulum stress (ERS) plays a pivotal role in the development of diabetes‐induced liver damage. Under elevated ERS, the unfolded protein response (UPR) balances cellular survival and death. There may be a preventive effect of herbal extracts against problems associated with diabetes. In this study, the effects of a hydroalcoholic extract from *Dorema aucheri* on liver damage, ERS, and UPR in diabetic rats were assessed.

**Methods:**

There were five groups of rats, each including six rats. A 60 mg/kg intraperitoneal injection of streptozotocin (STZ) was used to cause diabetes. Every day, 1 mL of normal saline was given to the control groups, who were normal and diabetic. For 28 days, three diabetic groups were given oral gavage doses of 250/500 mg/kg of *D. aucheri* extract and 500 mg/kg of metformin daily. Serum and liver samples were used to assess blood glucose, liver enzymes, oxidative stress, inflammatory markers, and antioxidant levels after treatment. Furthermore, liver tissue pathology, stereology, and gene expression were examined.

**Results:**

Compared to the diabetic groups, the treated groups exhibited a significant decrease in oxidative stress, inflammatory markers, liver enzymes, blood glucose, and increased antioxidant capacity. They significantly reduced mRNA levels of proapoptotic markers (*CHOP*, *caspase-9*, and *NF-κB*) and increased *GRP78*, *XBP1s*, and *BCL2* expression, suggesting attenuation of apoptosis and modulation of UPR‐related gene expression.

**Conclusion:**

*D. aucheri* extract may modulate UPR‐ and apoptosis‐related pathways, alleviate ERS, and potentially reduce diabetes‐associated complications in STZ‐induced diabetic rats. However, further mechanistic and translational studies are required to confirm these findings and evaluate their clinical relevance.

## 1. Introduction

Diabetes mellitus is a long‐term metabolic illness that causes high blood sugar and problems in the carbohydrate, lipid, and protein metabolism. People with chronic diabetes can get liver problems including nonalcoholic fatty liver disease, necrosis, fibrosis, and inflammation [1, 2]. In general, the key pathogenic pathways of the liver include oxidative stress, endoplasmic reticulum stress (ERS), chronic inflammation, unfolded protein response (UPR), autophagy, and cell death. These processes lead to pancreatic β‐cell failure and liver damage [3–5].

Oxidative stress is a key contributor to chronic diseases such as diabetes [6]. In diabetes, hyperglycemia‐induced oxidative stress plays a central role in liver damage [7, 8], driven by excessive free radical production through glucose oxidation, protein glycation, and subsequent degradation of glycated proteins [9, 10]. This imbalance between oxidants and antioxidants accelerates diabetic complications [11, 12]. Enhancing antioxidants such as glutathione peroxidase (GPX), glycosylated hemoglobin, and catalase (CAT), while reducing oxidants such as malondialdehyde (MDA) and carbonyl groups, can mitigate oxidative stress in diabetic patients [13]. On the other hand, oxidative stress also promotes interstitial inflammation by upregulating cytokines such as TNF‐α, IL1‐ß, and IL6 [14].

These inflammatory conditions exacerbate cellular stress and apoptosis through decreased expression of antiapoptotic proteins such as *BCL2*. In response, IL‐10 suppresses inflammatory signaling and may contribute to maintaining metabolic balance.

Another pathogenic mechanism involved in liver injury is endoplasmic reticulum (ER) stress. The ER is an intracellular membrane network responsible for protein synthesis, folding, and quality control. In diabetes, chronic hyperglycemia induces sustained ER stress in hepatocytes [15]. This condition results from the disruption of ER homeostasis, including impaired protein folding, accumulation of misfolded proteins, altered calcium balance, and redox imbalance [4, 16]. ERS plays a significant role in cellular dysfunction in diabetes, leading to the activation of adaptive protective mechanisms. To counteract ERS‐induced damage, cells initiate the UPR, a coordinated signaling network that aims at restoring ER homeostasis, primarily regulated by the ER chaperone *GRP78* (78‐kDa glucose–regulated protein). *GRP78* acts as a central regulator of the UPR by dissociating from and activating three major ER stress sensors: protein kinase RNA‐like endoplasmic reticulum kinase (*PERK*), inositol‐requiring enzyme 1 (*IRE1*), and activating transcription factor 6 (*ATF6*) [17–19].

*IRE1* and PERK primarily regulate the UPR by promoting adaptive gene expression and reducing protein synthesis. *IRE1* activates the splicing of *XBP1* mRNA to generate the active transcription factor *XBP1s*, while *PERK* attenuates global protein translation through the phosphorylation of *eIF2α*, leading to the selective translation of ATF4. Under moderate ER stress, these adaptive pathways enhance cell survival by reducing the protein‐folding burden and contributing to the restoration of ER homeostasis [20]. During prolonged or severe ER stress, apoptotic signaling is activated, largely through induction of CCAAT/enhancer‐binding protein homologous protein (*CHOP*), which is primarily upregulated via the *PERK–eIF2α–ATF4* signaling pathway [21]. *CHOP* triggers the innate apoptotic pathway by blocking B‐cell lymphoma 2 (*BCL2*), B‐cell lymphoma extra‐large (*BCL-XL*), and myeloid cell leukemia sequence 1 (*MCL-1*) and increasing the expression of the *BCL2*‐interacting mediator of cell death (BIM), which controls the mitochondrial outer membrane permeabilization mediated by BAX‐BAK [22]. Afterward, apoptotic components such as cytochrome c and members of the caspase cascade, such as caspase‐9, are released, resulting in the death of the cell [23]. ER stress is closely linked to the induction of proinflammatory signaling, partly mediated by *NF-κB* and AP‐1 activation downstream of UPR pathways, which contributes to inflammation and disturbances in glucose homeostasis [24]. Recent studies suggest that certain natural compounds, particularly plant extracts, may alleviate diabetes‐induced tissue damage in various organs [25–27]. Medicinal plants, with a long‐standing history of therapeutic use, remain among the most accessible options for managing diseases, especially metabolic disorders like diabetes. When used within safe limits and in accordance with medical guidelines, they are favored for their efficacy and relatively low incidence of side effects.

Medicinal plant extracts are rich in phenolic and flavonoid compounds, which possess antioxidant properties and can alleviate the effects of oxidative stress in diabetic patients [28, 29]. Research indicates that the herb *Dorema aucheri* is among the plants that may help reduce blood glucose and cholesterol levels in individuals with diabetes [30]. In fact, *D. aucheri*, belonging to the Apiaceae family, contains bioactive compounds such as flavonoids and coumarins, which contribute to its therapeutic potential. Flavonoids are recognized as antidiabetic agents and play a beneficial role in diabetes treatment [31]. Studies have shown that *D. aucheri* extract enhances the activity of antioxidant enzymes such as superoxide dismutase (SOD) and GPX, which are crucial for reducing oxidative stress induced by diabetes. This extract also increases serum concentrations of vitamin C, supporting its role in protecting liver cells from oxidative damage [32]. In another study, it was found that *D. aucheri* extract could restore the levels of liver enzymes such as SGPT, alkaline phosphatase, and SGOT to near‐normal levels in diabetic rats, indicating its potential for protecting liver function [30]. Additionally, in models of liver damage induced by carbon tetrachloride, *D. aucheri* extract significantly reduced the activity of liver enzymes such as alanine aminotransferase (ALT), aspartate transaminase (AST), and ALP [33].

Conversely, other studies have raised concerns regarding the effects of the *D. aucheri* extract. Some researchers have reported dose‐dependent cytotoxicity associated with *D. aucheri* extract [34]. These findings underscore the need for further investigation into the mechanisms and optimal dosage of *D. aucheri* extract for liver protection in diabetes. Notably, no study has yet explored the effects of its hydroalcoholic extract on the UPR signaling pathway. Therefore, this study aims to evaluate the protective effects of hydroalcoholic *D. aucheri* extract on liver histology and signaling pathways related to UPR, apoptosis, and antioxidant capacity in streptozotocin (STZ)‐induced diabetic rats.

## 2. Methods

### 2.1. Experimental Animals

At the start of the experiment, a total of 30 male Sprague‐Dawley rats (aged 2.5–3 months and weighing 250 ± 10 g) were acquired from the Laboratory Animals Research Center at Shiraz University of Medical Sciences in Iran. Before commencing the trials, the animals were used to the laboratory conditions for 2 weeks. The rats were used under laboratory conditions for 2 weeks, provided with rat food and water, and housed in stainless steel cages in a temperature‐controlled environment. The study protocols were approved by the Shiraz University of Medical Sciences Institutional Animal Ethics Committee (IR.SUMS.AEC.1401.050), in accordance with NIH Publication No. 85‐23, which provides guidelines for animal treatment and utilization. Also, the institutional animal ethics committee approved the study. Research animals were also cared for in accordance with ARRIVE standards.

### 2.2. Preparation of the Hydroalcoholic Extract From the *D. aucheri* Plant

We obtained specimens of *D. aucheri* from Zardband Pharmaceutical Company, Tehran, Iran. Leaves, flowers, and stems of *D. aucheri* were collected from around Yasuj (30°, 32′,54″ N, 51°, 42′, 51″ E, and an altitude of 1400 m), at flowering (end of June). Professor F. Attar identified and authenticated the plant, which was then deposited at the Central Herbarium of Tehran University (No. 46056TUH) [30, 34, 35]. We divided, cleansed, and allowed the aboveground portions of *D. aucheri* to dry naturally. Plant tissues weighing 100 g were ground and subjected to extraction using the percolation method with 500 mL of solvent. The plant tissues (100 g) were ground and subjected to extraction using the percolation method. This involved utilizing 500 mL of 70% ethanol at room temperature for 72 h. After the filtration process, a rotating device was used to evaporate ethanol at a temperature of 40°C. The solvent evaporation was performed using a vacuum desiccator for 24 h. Subsequently, the dried extract was stored at a temperature of −20^o^C, resulting in an efficiency of 16.5% [36].

### 2.3. Antioxidant Activity and Phenolic/Flavonoid Content of *D. aucheri*

#### 2.3.1. Antioxidant Activity (DPPH Assay)

The antioxidant capacity of the hydroalcoholic extract of *D. aucheri* was assessed using the DPPH free radical scavenging method. In brief, 20 μL of serially diluted plant extract was combined with 180 μL of 0.1 mM DPPH solution prepared in methanol and incubated in the dark at room temperature for 30 min. The reduction in absorbance was then recorded at 517 nm using a spectrophotometer, and the radical scavenging activity was calculated as a percentage. Quercetin served as the reference antioxidant [37].

#### 2.3.2. Total Phenolic Content

Total phenolic content was quantified using the Folin–Ciocalteu colorimetric assay. Briefly, 10 μL of different extract concentrations was added to 158 μL of distilled water, followed by 10 μL of Folin–Ciocalteu reagent. After a 9‐min reaction period, 30 μL of 7.5% sodium carbonate solution was introduced, and the mixture was incubated for 120 min. Absorbance was measured at 765 nm. Gallic acid was used as the calibration standard, and the results were expressed as mg gallic acid equivalents (GAEs) per gram of dry extract weight [37].

#### 2.3.3. Total Flavonoid Content

Total flavonoid content was determined using a colorimetric aluminum chloride method. In this procedure, 160 μL of water was mixed with 30 μL of diluted extract, 10 μL of 5% sodium nitrite, and 10 μL of 10% aluminum chloride solution. After incubation at room temperature for 60 min, 10 μL of 1 M sodium hydroxide was added, and the mixture was further incubated for 30 min. Absorbance was then read at 415 nm. Hesperidin was used as the standard, and flavonoid content was expressed as mg hesperidin equivalents (HEs) per gram of dry extract [38].

### 2.4. Inducing Diabetes and Monitoring Body Weight Following Therapy

The study involved injecting STZ (Co, Sigma, USA) at 60 mg/kg body weight into male rats overnight, administered intraperitoneally (i.p), using a citrate buffer with a pH of 4.5. Sprague‐Dawley rats were used in scientific experiments [39]. To assess the impact of STZ, we monitored the levels of blood glucose 1 week following the injection of STZ, regardless of whether the subjects had eaten or not. The rats’ tail veins were used to collect approximately 5 μL of whole‐blood samples using a lancet. The blood samples were then analyzed using an Accu‐Chek® glucometer. Blood glucose levels over 300 mg/dL were deemed the criteria for diagnosing diabetes. The body weights of rats were also assessed every week at 10 a.m.

### 2.5. Experimental Design

All rats were randomly assigned to five groups (*n* = 6 per group). The treatment period lasted 28 days. The hydroalcoholic extract of *D. aucheri* was administered daily by oral gavage at doses of 250 and 500 mg/kg [32]. The categorization of animals was as follows: The rats in Group I (control) were in good health and were free from diabetes. They orally received a 1 mL dose of normal saline. The diabetic rats in Group II (STZ) were given 1 mL of normal saline orally. The rats in Group III (STZ + metformin 500) received a dosage of 500 mg/kg of metformin. The rats in Group IV (STZ + *D. aucheri* 250) received a dosage of 250 mg/kg of *D. aucheri* extract. The rats in Group V (STZ + *D. aucheri* 500) were administered a dosage of 500 mg/kg of the extract.

Following the completion of the treatment period, we subjected the rats to a 12‐h fasting period and obtained roughly 5 mL of whole blood by heart puncture while the animals were under anesthesia. Initially, the rats were anaesthetized using a combination of ketamine and xylazine (100/10 mg/kg). After blood sampling and tissue isolation, following the ethical standards established by the university ethics committee, the animals were ultimately euthanized by placement in a CO_2_ chamber. The whole‐blood sample was centrifuged at a speed of 3500 revolutions per minute for 10 min to isolate the serum. The serum samples were stored in a sterile microcentrifuge tube at a temperature of −80^o^C until they were ready for further analysis.

### 2.6. Analysis of Serum Glucose, Liver Enzymes, and Lipid Profile

Diagnostic colorimetric kits (Biorex Fars) were used to measure serum glucose and albumin, while diagnostic colorimetric kits (Biosystem, Spain) were used to measure triglyceride, cholesterol, HDL, LDL, ALT, and AST. These measurements were performed using prestigious equipment (Hitachi, Japan).

### 2.7. Measurement of IL‐6 and IL‐1β Levels

The serum samples obtained from all the groups were produced following the instructions provided by the manufacturer in the kit. The Karmania Pars Gene Company’s ELISA kit was utilized to quantify the serum concentrations of inflammatory markers. The rat IL‐6 (CN: KPG‐RIL6) and IL‐1β (CN: KPG‐RIL1β) ELISAs utilize a solid‐phase sandwich ELISA method. This method involves a plate coated with a monoclonal antibody that specifically targets rat IL‐6 and IL‐1β.

### 2.8. Assessment of Oxidative Stress

The levels of SOD, CAT, GPX, and MDA in the serum were assessed using colorimetric kits obtained from the KIAZIST Company (Iran). In addition, liver tissue samples were disrupted in ice‐cold phosphate‐buffered saline and centrifuged with a force of 10,000 times the acceleration due to gravity for 15 min. The liquid portion was utilized to measure the total antioxidant capacity (TAC) and MDA levels by the colorimetric technique, employing kits obtained from Karmania Pars Gene Company (Iran).

### 2.9. RNA Isolation and cDNA Synthesis

Following the sacrifice of the rats, the liver tissues were submerged in an RNA solution (SinaClon) for 24 h and then stored at −80°C. The RNX‐PLUS or TRIzol isolation reagent, as recommended by the manufacturer (SinaClon), was used to extract total RNA. Subsequently, the Nanodrop (Thermo Fisher Scientific, Waltham, MA, USA) was used to determine the extracted RNA yield and purity. Only the samples with ratios greater than 1.8 were selected for cDNA synthesis. Ultimately, a total of 30 μL was used to create cDNA using the Danesh Bonyan (Tech) Reverse Transcription Kit (SinaClon), with just 1 μg of RNA. The cDNAs were retained for subsequent utilization in quantitative real‐time polymerase chain reaction (qRT‐PCR).

### 2.10. Evaluation of the *GRP78*, *XBP1s*, *NF-κB*, *CHOP*, *BCL2*, and *Caspase-9* mRNA Expression in the Liver by qRT‐PCR

The qRT‐PCR reaction consisted of 10 μL of the SYBR Green DNA qRT‐PCR Master Mix (Amplicon), 0.8 μL of each primer (10 pmol), 7.6 μL of dH2O, and 0.8 μL of cDNA, for a total volume of 20 μL. Table 1 presents the qRT‐PCR primers employed in the experiment. The qRT‐PCR procedure involved an initial step of heating the sample to 95°C for 10 min, followed by 40 cycles of 15 s at 95°C and subsequent annealing at a specified temperature, such as 60°C for 30 s and 72°C for 30 s. The process concluded with a 10‐min elongation step at 72°C. The results were subjected to electrophoresis on a 1.5% (w/v) agarose gel and then seen using gel red staining.

**TABLE 1:** The sequence of designed primers.

Gene description	Gene id	Sequences (5′‐> 3′)	Product size (bp)
*BCL2*: Sense	NM_016993.1	GGAGGATTGTGGCCTTCTTT	100
*BCL2*: Antisense		GTCATCCACAGAGCGATGTT	

*Caspase-9*: Sense	NM_031632.1	ACATCTTCAATGGGACCGGC	85
*Caspase-9*: Antisense		TCTTTCTGCTCACCACCACAG	

*GRP78*: Sense	NM_013083.2	CAGCCCACCGTAACAATCAAG	186
*GRP78*: Antisense		TCCTGTCCCTTTGTCTTCAGC	

*XBP1s*: Sense	NM_173149.2	GGACACGCTTGGGGATGAATG	214
*XBP1s*: Antisense		CTGCACCTGCTGCGGACT	

*CHOP*: Sense	NM_001109986.1	TACACCACCACACCTGAAAGC	216
*CHOP*: Antisense		GCAGGGTCAAGAGTAGTGAAG	

*NF-κB*: Sense	NM_001415012.1	CCAGCACCAAGACCGAAGCAA	170
*NF-κB*: Antisense		CGCCAGCAGCATCTTCACATC	

*β-Actin*: Sense	NM_017008.4	CACACCCGCCACCAGTTCG	100
*β-Actin*: Antisense		ACCCATTCCCACCATCACAC	

### 2.11. Quantitative RT‐PCR

The liver expression levels of *caspase-9*, *BCL2*, *GRP78*, *XBP1s*, *NF-κB*, and *CHOP* were assessed using the ABI real‐time PCR 7500 system via qRT‐PCR analysis. The expression levels of the *GRP78*, *XBP1s*, *NF-κB*, *CHOP*, *BCL2*, and *caspase-9* transcripts were compared to rat βactin, which served as the housekeeping gene.

### 2.12. Stereological Study

At the end of the study, the liver weight was recorded, and the initial liver volume (V primary) was measured by the Scherle method [40, 41]. To obtain isotropic uniform random (IUR) sections, the orientator method was applied. From each liver, 8–12 tissue slabs were systematically sampled. A circular tissue punch (trocar) was taken from one slab per liver to assess shrinkage. All slabs and circular punches were embedded in paraffin, sectioned at 5 μm (for volume fraction estimation) and 25 μm (for cell counting using the dissector method), stained with hematoxylin–eosin and Masson’s trichrome, and coverslipped.

The degree of shrinkage (DSh) was calculated using the following formula:

(1) DSh=1−AAAB1.5,

where *A* *A* and *A* *B* are the areas of the circular piece after and before processing and staining. Total final volume of the liver was calculated by the following formula:

(2) Vfinal=1−DSh×Vprimary.

### 2.13. Estimation of Liver Structure via a Stereological Study in the Different Experimental Groups

**FIGURE 1:**
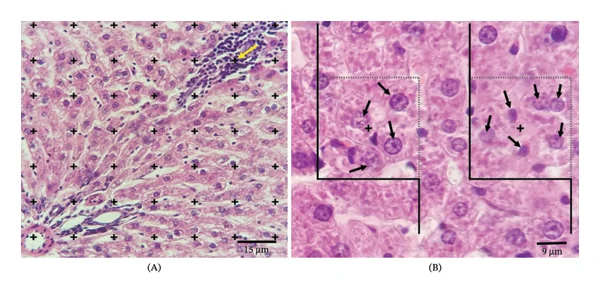
Liver sampling methods for stereological parameter evaluation. (A) Volume density estimation: The volume density of distinct liver structures was assessed using a point‐counting method. Only points that fell on the upper‐right corner of each target’s cross‐hair intersection were included in the analysis (indicated by yellow arrows). (B) Nuclei counting: An optical dissector approach was employed, applying an unbiased counting frame to determine the total number of hepatocyte nuclei.

Volume density (Vv) of each structure (hepatocytes, portal triads, sinusoids, central veins, necrotic and fibrotic tissue, inflammatory foci) was estimated using the point‐counting method on a uniform random grid according to the following formula (Figure 1A):

(3) Vvstructure=∑i=1npstructure∑i=1nreference,

where “Σ*P* structure” is the total number of points hitting the structure of interest, and “Σ*P* reference” is the total number of points hitting the whole liver section. The absolute volume of each structure was then

(4) Vstructure=Vvstructure/liver×Vfinal.

### 2.14. The Total Number of Hepatocyte Nuclei

To estimate the total number of hepatocyte nuclei, sections with 25 mm thickness were used. By means of the stereology software, an unbiased counting frame was superimposed on the images of the liver sections viewed on the monitor. The position of the microscopic field was selected by systematic random sampling. A high numerical aperture 100‐magnification (NA 1.4) oil immersion lens was used. The number of hepatocytes’ nuclei was estimated using the dissector method. Dissector is a method in which the cells are selected with uniform random probability, free from the assumption of size and shape. By an optical dissector, the cells are sampled into thick microscopic sections observed with a microscope. An unbiased counting frame with inclusion (right and upper) and exclusion (left and lower) borders was superimposed on the images. This frame avoids the edge effect and biased counting of the particles, and all of the nuclei profiles, regardless of their shape, are counted by the frame and have the same probability of being sampled. Several fields, using the objective of × 100, from all of the 25‐μm‐thick sections were selected and a microcator (ND 221 B, Heidenhain, Germany) was used for counting the numerical density (Nv), or the number of the nucleus in the unit volume of the hepatocytes was estimated using the number of cell count coming into focus, using the following formula (Figure 1B):

(5) Nv=∑i=1nQ∑i=1nP×h×a/f×tBA,

where ∑*A* is the total area of the unbiased counting frame in all fields, *h* is the optical height of the dissector, where Σ*Q* is the number of the whole nucleus counted in all the dissectors, a/frame is the area of the counting frame, Σp is the total number of the counted frames, *BA* is the microtome block advance to cut the block, and *t* is the mean of the final section thickness.

### 2.15. Statistical Analysis

Statistical analyses were performed using IBM SPSS Statistics for Windows, Version 22.0 (IBM Corp., Armonk, NY, USA). All data are presented as mean ± standard deviation (SD). The normality of data distribution was assessed using the Shapiro–Wilk test. Homogeneity of variances across the groups was examined using Levene’s test. In all cases, the assumptions of normality and homoscedasticity were met. The comparison of biochemical parameters across groups was examined using one‐way analysis of variance (ANOVA). After obtaining statistically significant findings from the ANOVA with a *p* value of less than 0.05, Tukey’s post hoc analysis was performed. A statistical significance level of *p* < 0.05 was established as the threshold to evaluate the significance.

## 3. Results

### 3.1. Antioxidant Activity and Phenolic/Flavonoid Content of *D. aucheri*

The hydroalcoholic extract of *D. aucheri* exhibited antioxidant activity in the DPPH radical scavenging assay, with an IC_50_ value of 0.33 ± 0.09 mg/ML (Table 2). This relatively low IC_50_ value indicates strong free radical scavenging potential, as lower IC_50_ values reflect higher antioxidant potency. In comparison, the reference compound quercetin showed an IC_50_ value of 18.56 ± 2.19 μM.

**TABLE 2:** Evaluation of total flavonoid, total phenolic, and DPPH in the extract of *Dorema aucheri*.

Name	DPPH (mg/mL)	Total flavonoid (mg/g of dry extract)	Total phenolic (mg/g of dry extract)
*Dorema aucheri*	0.33 ± 0.09	33.98 ± 1.69	189.45 ± 4.24

*Note:* Data were expressed as mean ± SD.

The total phenolic content of the extract was determined to be 189.45 ± 4.24 mg/g of dry extract. Given the well‐established antioxidant properties of phenolic compounds, this relatively high concentration suggests that phenolics are likely major contributors to the observed antioxidant activity.

In addition, the total flavonoid content was measured at 33.98 ± 1.69 mg/g of dry extract. Although lower than the phenolic fraction, flavonoids may also contribute to the overall antioxidant capacity of the extract through their free radical scavenging properties.

### 3.2. The *D. aucheri* Extract Influences the Body Weight, Fasting Blood Sugar (FBS), Lipid Profile, and Liver Enzymes

A significant increase in the levels of FBS, AST, ALT (*p* < 0.001), cholesterol, and HDL (*p* < 0.01) was detected in diabetic rats as compared to the control rats. Additionally, a significant decrease in body weight (*p* < 0.001) was observed in diabetic rats. However, there was no significant increase in TG and LDL levels, nor a significant decrease in Alb levels, in diabetic rats compared to control rats. Administering *D. aucheri* extract and metformin to diabetic rats resulted in a substantial reduction in the FBS level (*p* < 0.001), AST (*p* < 0.01), and ALT activities (*p* < 0.001). Additionally, it led to a rise in the body weight growth, bringing it close to the values observed in the control group (*p* < 0.001) at both doses of 250 and 500 mg. The administration of *D. aucheri* and metformin to diabetic rats did not result in a significant change in albumin levels. As the dosage of *D. aucheri* extract (500 mg) increased, the blood lipid profile of the treated individuals exhibited a restoration of TG, cholesterol, HDL‐C, and LDL‐C levels to a similar extent as those observed in the control group. Nevertheless, these levels did not reach statistical significance. If metformin therapy led to a noteworthy rise in TG (*p* < 0.05) and (*p* < 0.01) cholesterol levels in diabetic rats compared to control rats, the increase in HDL and LDL was not statistically significant (Table 3).

**TABLE 3:** Biochemical parameters in the treated groups.

Variants	Control	STZ (60 mg/kg)	Metformin (500 mg/kg)	*D. aucheri* (250 mg/kg)	*D. aucheri* (500 mg/kg)
Body weight (g)	312.1 ± 23.9^†††^	183.9 ± 24.7^∗∗∗^	215.3 ± 18.4^∗∗∗^	212.8 ± 31.9^∗∗∗^	191.4 ± 17.7^∗∗∗^
FBS (mg/dL)	157.7 ± 16.5	385.8 ± 52.2^∗∗∗^	301.3 ± 26.2^†∗∗∗^	293.4 ± 84.6^†∗∗∗^	299.6 ± 81.3^†∗∗∗^
TG (mg/dL)	63.8 ± 30.1	74.2 ± 38.0	182.6 ± 114.3^†∗^	124.6 ± 95.9	65.0 ± 31.8
Chol (mg/dL)	39.0 ± 6.7	55.0 ± 9.7^∗∗^	57.0 ± 9.5^∗∗^	49.4 ± 9.1	42.7 ± 13.4
HDL (mg/dL)	16.9 ± 3.1	25.0 ± 6.5^∗∗^	22.0 ± 3.7	19.3 ± 4.4	19.00 ± 5.6
LDL (mg/dL)	6.0 ± 1.0	6.1 ± 1.6	7.7 ± 2.7	7.0 ± 2.7	5.9 ± 1.9
ALT (U/L)	178.4 ± 35.1	476.7 ± 242.5^∗∗∗^	295.9 ± 37.7^††^	263.7 ± 68.2^†††^	255.9 ± 76.5^†††^
AST (U/L)	119.5 ± 18.2	287.8 ± 128.9^∗∗∗^	152.0 ± 36.9^†^	160.0 ± 38.9^††^	137.7 ± 61.6^††^
Alb (g/dL)	2.5 ± 0.3	2.1 ± 0.5	2.00 ± 0.3	2.03 ± 0.3	2.00 ± 0.4

*Note:* Data are presented as mean ± SD (*n* = 6). ^∗^ *p* < 0.05, ^∗∗^ *p* < 0.01, ^∗∗∗^ *p* < 0.001 compared to the control group. ^†^ *p* < 0.05, ^††^ *p* < 0.01, ^†††^ *p* < 0.001 compared to the STZ group.

### 3.3. The Impact of *D. aucheri* Extract on the Levels of Inflammatory Markers in the Serum

Table 4 displays the concentrations of inflammatory markers, specifically IL‐6 and IL‐1β, in the serum of both experimental and normal rats. Diabetes led to a notable increase in IL‐6 and IL‐1β levels. In comparison with the control group, the experimental groups showed significantly different results (*p* < 0.001, *p* < 0.01, and *p* < 0.001, respectively). The *D. aucheri* extract and metformin separately led to a significant reduction in IL‐6 and IL‐1β levels in diabetic rats. The impact was more prominent in the group of rats treated with *D. aucheri* extract, with a statistical significance at *p* < 0.01 for IL‐6 and *p* < 0.001 for IL‐1β.

**TABLE 4:** Serum inflammatory markers in the treated groups.

Variants (pg/mL)	Control	STZ (60 mg/kg)	Metformin (500 mg/kg)	*D. aucheri* (250 mg/kg)	*D. aucheri* (500 mg/kg)
IL‐6	5.3 ± 0.9	14.0 ± 2.0^∗∗∗^	9.2 ± 1.0^†^	6.8 ± 2.0^††^	8.5 ± 1.2^††^
IL‐1β	8.1 ± 1.4	22.6 ± 2.0^∗∗∗^	18.2 ± 1.8^††∗∗∗^	13.2 ± 1.8^†††∗∗∗^	13.5 ± 2.0^†††∗∗∗^

*Note:* Data are presented as mean ± SD (*n* = 6). ^∗∗∗^ *p* < 0.001 compared to the control group. ^†^ *p* < 0.05, ^††^ *p* < 0.01, ^†††^ *p* < 0.001 compared to the STZ group.

### 3.4. *D. aucheri* Extract has Been Found to Affect the Serum Levels of Antioxidant and Oxidant Markers

Diabetes was associated with a significant decrease in the serum GPx (*p* < 0.01), CAT (*p* < 0.01), SOD (*p* < 0.001) activities, and an increase in MDA (*p* < 0.001) level. In the group of diabetic rats that were treated with *D. aucheri extract* and metformin, MDA levels dropped significantly, while GPx (without metformin), CAT, and SOD activities went up slightly. The group of rats treated with 250 mg of *D. aucheri* extract showed a greater effect on GPx, CAT, and SOD activities (*p* < 0.001), (*p* < 0.01), and (*p* < 0.01), respectively (Table 5).

**TABLE 5:** Serum level of antioxidant/oxidant markers in the treated groups.

Variants	Control	STZ (60 mg/kg)	Metformin (500 mg/kg)	*D. aucheri* (250 mg/kg)	*D. aucheri* (500 mg/kg)
MDA (nmol/mL)	164.7 ± 11.5	373.5 ± 36.5^∗∗∗^	199.6 ± 10.7^†††^	234.2 ± 17.3^∗†††^	259.1 ± 32.9^∗∗††^
GPX (mIU/mL)	7.5 ± 2.1	3.6 ± 0.8^∗∗^	6.0 ± 1.1	8.8 ± 1.3^†††^	7.4 ± 0.8^††^
Catalase (mIU/mL)	564.7 ± 86.9	275.6 ± 52.3^∗∗^	547.0 ± 114.3^†^	629.7 ± 198.4^††^	527.8 ± 77.2^†^
SOD (IU/mL)	6.1 ± 2.0	1.1 ± 0.5^∗∗∗^	3.7 ± 0.8^†^	4.97 ± 1.7^††^	4.0 ± 1.2^†^

*Note:* Data are presented as mean ± SD (*n* = 6). ^∗^ *p* < 0.05, ^∗∗^ *p* < 0.01, ^∗∗∗^ *p* < 0.001 compared to the control group. ^†^ *p* < 0.05, ^††^ *p* < 0.01, ^†††^ *p* < 0.001 compared to the STZ group.

### 3.5. The Extract of *D. aucheri* Regulates the Levels of MDA and TAC Parameters in Liver Tissue

The levels of MDA and TAC in the liver tissue of experimental and normal rats are displayed in Table 6. When compared to the matching control group, there was a substantial increase in the MDA concentration and a significant decrease in the TAC concentration in the liver tissues during diabetes (*p* < 0.01). It was found that treating diabetic rats with *D. aucheri* extract and metformin increased (*p* < 0.05) the amount of the TAC in the liver tissue and decreased (*p* < 0.01) the amount of MDA. All three groups treated with metformin at doses of 250 and 500 mg of *D. aucheri extract* showed this effect.

**TABLE 6:** Level of MDA and TAC parameters in the liver tissue of the treated groups.

Variants (nmol/mg protein)	Control	STZ (60 mg/kg)	Metformin (500 mg/kg)	*D. aucheri* (250 mg/kg)	*D. aucheri* (500 mg/kg)
MDA	0.25 ± 0.05	1.4 ± 0.5^∗∗^	0.3 ± 0.1^††^	0.2 ± 0.2^††^	0.3 ± 0.2^††^
TAC	50.6 ± 15.1	16.1 ± 3.2^∗∗^	49.6 ± 10.3^†^	47.0 ± 7.5^†^	49.5 ± 7.7^†^

*Note:* Data are presented as mean ± SD (*n* = 6). ^∗∗^ *p* < 0.01 compared to the control group. ^†^ *p* < 0.05, ^††^ *p* < 0.01, ^†††^ *p* < 0.001 compared to the STZ group.

### 3.6. The Extract of *D. aucheri* has an Impact on the Expression Levels of ER Stress‐Related Genes in the Liver, as Well as on the *NF-κB*

**FIGURE 2:**
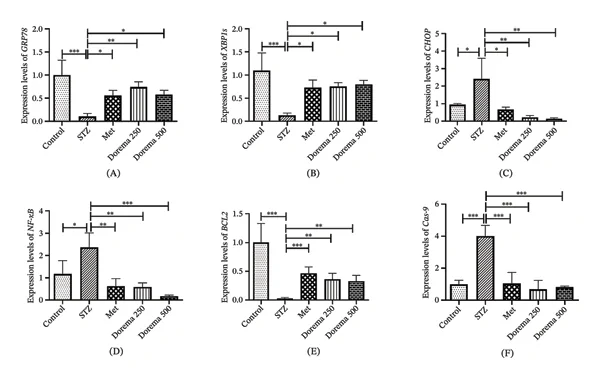
Level of gene expression in liver tissue of treated groups. (A) GRP78, (B) XBP1s, (C) CHOP, (D) NF‐κB, (E) BCL2, and (F) CASP‐9. All values are presented as mean ± SD. (^∗^ *p* < 0.05; ^∗∗^ *p* < 0.01; ^∗∗∗^ *p* < 0.001).

Figure 2 depicts the concentrations of *GRP78*, XBP‐1, *BCL2*, *CHOP*, *NF-κB*, and CASP‐9 in the liver tissue of rats during the experiment. During diabetes, there was a significant increase in the levels of *CHOP* (*p* < 0.05), *NF-κB* (*p* < 0.05), and CASP‐9 (*p* < 0.001) in the liver tissue, while there was a significant drop in the levels of *GRP78* (*p* < 0.001), XBP‐1 (*p* < 0.001), and *BCL2* (*p* < 0.001). Administration of the *D. aucheri* extract and metformin to diabetic rats resulted in a substantial decrease in the levels of *CHOP*, *NF-κB*, and CASP‐9, while there was a significant increase in the levels of *GRP78*, XBP‐1, and *BCL2*. The rats that received a dosage of 500 mg of *D. aucheri* extract exhibited a more pronounced impact on *NF-κB* (*p* < 0.001), whereas those that received a dosage of 250 mg of *D. aucheri* extract exhibited a more pronounced impact on *GRP78* (*p* < 0.01). Both the 250 and 500 mg doses of *D. aucheri* extract exhibited statistically significant effects on XBP‐1 (*p* < 0.05), *BCL2* (*p* < 0.01), *CHOP* (*p* < 0.01), and CASP‐9 (*p* < 0.001).

### 3.7. Analysis of Liver Tissue Structures Reveals Stereological Observations About the Effects of *D. aucheri* Extract

**FIGURE 3:**
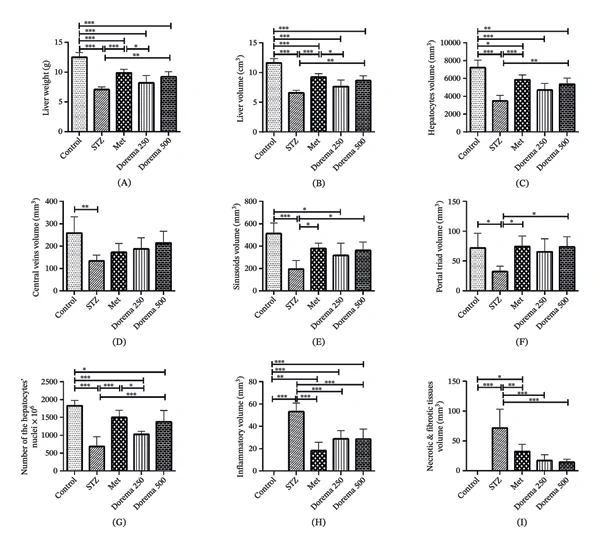
Stereology liver tissue of the treated groups. (A) Liver weight, (B) liver volume, (C) hepatocytes volume, (D) central veins volume, (E) sinusoids volume, (F) portal triad volume, (G) number of the hepatocytes nuclei x106, (H) inflammatory volume, and (I) necrotic and fibrotic tissues volume. All values are presented as mean ± SD. (^∗^ *p* < 0.05; ^∗∗^ *p* < 0.01; ^∗∗∗^ *p* < 0.001).

The diabetic rats exhibited a notable increase in the quantity of necrotic and fibrotic tissues as compared to the control group (Figure 3). Simultaneously, there was a considerable decline in the liver tissues, liver weight, liver volume, hepatocytes, central veins, sinusoids, the portal triad, and the number of hepatocytes. In the group of rats with diabetes, the administration of *D. aucheri* extract and metformin resulted in a significant augmentation in the liver weight and volume, hepatocyte volume, central vein volume, sinusoid volume, portal triad volume, and hepatocyte count. In addition, it led to a decrease in the amount of inflammatory, necrotic, and fibrotic tissues. The STZ group exhibited a substantial effect on the liver weight and inflammatory volume in comparison with the control group. Nevertheless, there was no notable impact on the volume of the central vein in any of the three groups that were administered either 250 or 500 mg of *D. aucheri extract* or metformin. The administration of metformin and a 500‐mg dose of *D. aucheri* resulted in a notable impact on the liver volume, portal triad volume, number of hepatocytes, and hepatocyte volume in comparison with the group of diabetic rats. However, the administration of the 250‐mg dose of the *D. aucheri* group did not have a notable impact on these parameters. The impact on the sinusoid volume was more pronounced in the groups administered with 250 and 500 mg of *D. aucheri*, but not in the groups treated with metformin. This was in contrast to the group of rats with diabetes. The administration of metformin had a significant effect on the amount of necrotic and fibrotic tissue in comparison with the group of rats with diabetes. Nevertheless, the groups that received doses of 250 and 500 mg did not exhibit a significant impact.

### 3.8. Induced Histological Alterations in the Liver Tissue by *D. aucheri* Extract

**FIGURE 4:**
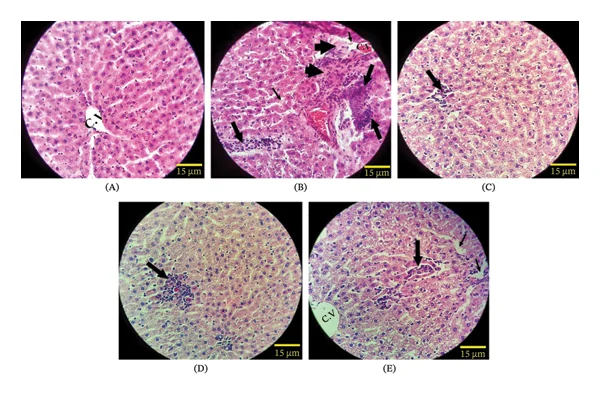
Histopathological evaluation of the rats’ liver. (A) Control group; (B) STZ group; (C) metformin (500 mg/kg) group; (D) and (E) *D. aucheri*–treated rats (250 and 500 mg/kg) (hematoxylin–eosin staining, 400x magnification).

The control sample’s tissue exhibits no discernible alterations in its visual characteristics, as illustrated in Figure 4A, and the general arrangement and coherence of the tissue structure remain intact. The cytoplasm of the hepatocytes displays a clear acidophilic property, while the nucleus is characterized by euchromatin. The quantity of Kupffer cells in the sinusoidal region is sufficient, and there is no discernible edema or buildup.

Figure 4B shows that the STZ group had an increased amount of connective tissue and signs of tissue deterioration, making it challenging to determine how the cells are related in the samples.

Within specific areas, the tissue exhibits fractured sinusoidal gaps (shown by thin arrows). There are evident accumulations of blood cells in the sinusoidal area, and there is a considerable rise in the number of inflammatory cells (shown by a thick arrow). The hepatocytes display an atypical morphology, characterized by opaque and black nuclei, as well as an expansion of the vacuole space both internally and externally to the cells. The samples from the diabetic group contain hepatocytes that exhibit either two nuclei or no nuclei. The central lobular vein (C.V.) displays blood accumulation and does not have a clearly defined endothelium. Figure 4C displays evidence of the healing process in the samples taken from the diabetic group treated with metformin. Hepatocytes retain the vacuolar cytoplasmic space, albeit its intensity is reduced compared to the diabetes group. There is no gradual increase, and the sinusoidal space is appropriately sized. While the number of inflammatory cells in the region has decreased, they remain observable in specific regions (shown by the thick arrow). There are no abnormal blood accumulations or indications of bleeding, and the tissue arrangement and consistency are optimal. The liver tissue of the diabetic group, at a dosage of 250 mg/kg of the hydroalcoholic extract from the *D. aucheri* plant, has satisfactory organization and consistency. Sinusoidal spaces do not typically exhibit extreme expansion or odd dimensions. Despite the repeated migration of the hepatocytes away from the degenerative process, vacuoles remain readily apparent in their cytoplasm. The sinusoidal area also shows a significant accumulation of inflammatory cells (thick arrow) (Figure 4D). Conversely, most of the tissue areas in the diabetic group that were administered a 500 mg/kg dosage of the hydroalcoholic extract derived from the *D. aucheri* plant exhibited typical characteristics. Hepatocytes commonly reveal intact cytoplasm and nucleus without any sign of degeneration. The lobule of the liver exhibits a certain size and form. Both the C.V. and the sinusoidal space are in good condition. Nevertheless, specific regions still exhibit the presence of slightly inflammatory cells (Figure 4E).

**FIGURE 5:**
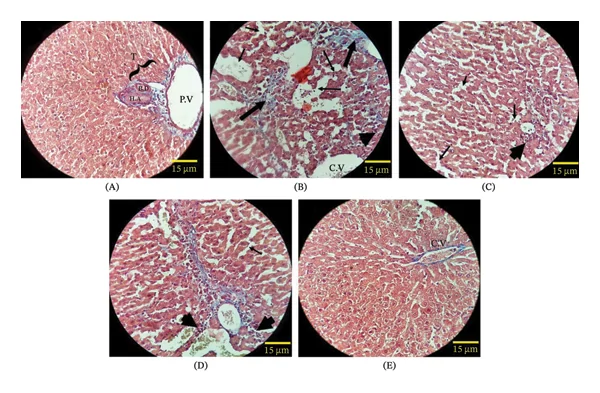
Histopathological investigation of the effect of the hydroalcoholic extract of the *D. aucheri* plant on the structure of the liver tissue in diabetic rats. (A) control group; (B) STZ group; (C) metformin (500 mg/kg) group; and (D and E) *D. aucheri*‐treated rats (250 and 500 mg/kg). Campbell’s triad space (T), portal vein (P.V.), hepatic artery (H.A.), and bile duct (B.D.) were examined (Mason’s trichrome staining, 400x magnification).

Figure 5A illustrates that in the control group, the triad space (T), portal vein (P.V.), hepatic artery (H.A.), and bile duct (B.D.) of Campbell exhibit normal characteristics. Furthermore, the tissue space remains structurally sound and well‐arranged. The hepatocytes arrange themselves in elongated columns that extend toward the C.V. The liver tissue samples from the STZ group display fibrosis, as indicated by the thick arrow, and necrosis in specific areas. The samples demonstrate a lack of cellular cohesion and display regions of tissue disintegration, leading to a significant increase in sinusoidal gaps, as illustrated by the thin arrow. The sinusoidal region exhibits conspicuous aggregations of blood cells, accompanied by a significant increase in the quantity of inflammatory cells (shown by the arrow tip). The hepatocytes have anomalous morphology, characterized by an enlargement of the vacuole space both internally and externally to the cells (Figure 5B). The diabetic group administered a dosage of 500 mg/kg of metformin had visible indications of the healing process, as seen in Figure 5C. Although hepatocytes still display vacuolar cytoplasmic space, their intensity has decreased compared to the diabetic group. A slender arrow denotes a subtle enlargement of the sinusoidal cavity. In the given area, the number of inflammatory cells has decreased, while there are still some instances of scattering in specific regions (shown by the arrow tip). Nevertheless, the group of individuals with diabetes who were administered the hydroalcoholic extract of the *D. aucheri* plant at a dose of 250 mg/kg still encountered damage to their liver tissue, displaying areas of fibrosis in particular sites. Hepatocytes are often extracted throughout the degenerative process although their cytoplasm still exhibits distinct vacuoles. The arrowhead in Figure 5D signifies the existence of inflammatory cells, which invade the region. Most tissue sections in the diabetic group that received the hydroalcoholic extract of the *D. aucheri* plant at a dosage of 500 mg/kg (as shown in Figure 5E) exhibited a normal appearance. The hepatocytes showed no evidence of degradation and consistently revealed typical cytoplasm and nucleus. The hepatic lobule has a characteristic size and features. The sinusoidal space and the C.V. appear to be within normal limits; nonetheless, there is evidence of modest infiltration of inflammatory cells in specific regions.

## 4. Discussion

Diabetes mellitus is a serious condition that can cause harmful consequences in numerous individuals [42]. Extensive research has been conducted in the field of therapy, leading to the development of pharmaceuticals [43]. However, herbal medicines have an advantage due to their reduced complications and adverse effects. Moreover, there are growing data suggesting that herbal medicine could potentially be used as a treatment for diabetes mellitus [44].

This animal study examined the impact of hydroalcoholic extract from *D. aucheri*, which is abundant in flavonoids renowned for their antioxidant characteristics, on liver injury in rats with diabetes caused by STZ. We orally fed *D. aucheri* extract through gavage at doses of 250 and 500 mg/kg for 28 days. Based on our extensive research, there is limited knowledge regarding the mechanism of action of *D. aucheri*. Consequently, our main objective was to investigate the mRNA expression of UPR‐related genes, which could provide insight into the possible gene expression–based evidence of action of this medication on liver cells. Ultimately, the effectiveness of this extract was confirmed by evaluating the stereological characteristics.

In our experiment, a remarkable increase in blood glucose and a reduction in body weight were observed in STZ‐induced diabetic rats compared with the healthy control group. STZ‐induced diabetes leads to a fall in body weight caused by increased breakdown of tissue proteins and a decrease in muscle mass due to inadequate levels of insulin [45]. Our research showed that giving diabetic rats the *D. aucheri* extract led to a substantial reduction in high blood sugar levels and a notable rise in body weight compared to diabetic rats that did not receive the extract. These results are consistent with the findings of Ahangarpour et al. [30]. In line with our findings, Satheesh and Pari discovered that the administration of metformin (500 mg/kg/day) effectively reduced blood glucose levels in diabetic rats, who were utilized as the positive control group [46]. Furthermore, the efficacy of *D. aucheri* extract in lowering glucose levels was almost superior to that of administering metformin (500 mg/kg). To our knowledge, no previous study has directly compared the hypoglycemic effects of *D. aucheri* and metformin.

Regarding the lipid profile, the production of diabetes in rats with STZ resulted in an elevation of serum cholesterol, HDL, LDL, and triglycerides, which aligns with the findings published by Watcho et al. [47]. The extract administered at a dose of 500 resulted in a reduction in serum lipid levels in diabetic rats. This outcome aligns with the findings of Nahvinejad and colleagues [48]. The efficacy of this decrease surpassed that of metformin treatment. It is commonly recognized that people with diabetes often suffer from hyperlipidemia. Reduced insulin levels can result in the inability to activate lipoprotein lipase, leading to an increase in the breakdown of fats, high levels of triglycerides in the blood, and an elevated concentration of free fatty acids in the plasma [49, 50].

Although metformin is well known for improving glycemic control and is generally considered neutral or beneficial with respect to lipid metabolism, in the present study, triglyceride and cholesterol levels were higher in the metformin‐treated group compared with the untreated diabetic (STZ) group. At first glance, this may appear unexpected; however, it can be better interpreted in the context of the severe insulin‐deficient state induced by STZ (60 mg/kg), in which pancreatic β‐cell destruction leads to persistent and profound metabolic dysregulation. In this model, dyslipidemia is primarily driven by impaired insulin signaling, reduced lipoprotein lipase activity, and increased hepatic lipid output, leading to sustained disturbances in lipid clearance and transport. Under such conditions, partial improvement in glycemia may not be sufficient to restore normal lipid homeostasis. Consistently, fasting blood glucose remained markedly elevated in the metformin‐treated group, indicating incomplete metabolic control. Therefore, the observed increases in triglycerides and cholesterol are more likely reflective of ongoing diabetic dysmetabolism rather than a direct effect of metformin. These findings highlight the context‐dependent nature of metabolic effects of metformin and suggest that its impact on lipid parameters may be limited in severe insulin‐deficient states such as STZ‐induced diabetes. Further investigations may help clarify these lipid responses, considering factors such as treatment duration, dose, and the severity of the diabetic model.

Patients with diabetes mellitus experience elevated levels of AST and ALT [51]. Our findings indicate that the levels of these two enzymes are higher in STZ‐induced diabetic rats compared to the control group. The observed decline in AST and ALT levels in the treatment groups indicates that the *D. aucheri* extract had a hepatoprotective impact on the liver function. A previous study yielded comparable findings [30]. Activated Kupffer cells produce reactive oxygen species (ROS), such as superoxide anions, hydrogen peroxide, and hydroxyl radicals. These radicals are recognized as a significant contributor to liver damage, like the effects observed in diabetes. It will trigger the release of inflammatory cytokines [8, 52]. The liver contains potent antioxidants such as SOD, CAT, and the glutathione (GSH) enzyme family, which encompasses glutathione‐S‐transferases (GSTs) and GPXs. These antioxidants can both counteract free radicals and protect liver cells from oxidative damage [53]. Prior research has demonstrated that in the presence of high blood sugar levels (hyperglycemia), the functioning of antioxidant enzymes such as SOD, CAT, and GPXs reduces. This reduction in enzyme activity results in the buildup of ROS, leading to oxidative damage in the liver [54, 55]. In addition, lipid peroxidation takes place when ROS, also known as free radicals, are activated. Diabetes leads to elevated MDA levels, which are a result of lipid peroxidation [56]. Sharma et al.’s study found that induced hyperglycemia in rats led to a reduction in TAC [57]. The current study observed an increase in the serum levels of IL‐6, IL‐1β, and IL‐10 coinciding with the development of diabetes in rats. This rise indicates the onset of inflammation and the increased secretion of IL‐6 and IL‐1β as proinflammatory cytokines, along with IL‐10 as an anti‐inflammatory cytokine. These findings are consistent with previous research that suggests diabetes can initiate inflammatory reactions [58, 59]. The concentrations of IL‐6, IL‐1β, and IL‐10 in the bloodstream of rats administered the *D. aucheri* extract for a duration of 28 days showed a significant reduction compared to the diabetic group. The efficacy of this decrease surpassed that of metformin treatment. In addition, Nahvinejad et al.’s investigation found a rise in the levels of SOD, CAT, and GPXs enzymes, as well as an increase in the concentration of TAC [48]. The observed results can be attributed to the plant’s antioxidant characteristics. Moreover, our results showed a decrease in MDA levels after administering *D. aucheri* extract, which aligns with the findings of Raeissi et al., who examined the impact of this extract on individuals with diabetes [60].

ERS is a condition where proteins are misfolded and accumulated within the ER membrane, leading to liver‐related diseases [19, 61]. Therefore, it can serve as a significant route in liver‐related conditions such as diabetes mellitus. The ER stress triggers the activation of the IRE1 branch of the UPR pathway, which subsequently leads to the activation of X‐box–binding protein 1 (XBP‐1). During this inquiry, we found a notable decrease in the hepatic gene expression of the UPR markers, *GRP78* and XBP‐1, in the diabetic rats as compared to the healthy control group. This finding supports the findings of the Afrin et al.’s study conducted in Japan [62]. Both markers exhibited a substantial rise following the administration of *D. aucheri* extract. Induction of *CHOP* triggers apoptotic pathways in cases of prolonged and severe ERS [21]. *CHOP* induces the intrinsic apoptotic pathway, which triggers the liberation of apoptotic molecules, including cytochrome c and the activation of the caspase cascade, specifically *caspase-9*, ultimately leading to cell death [22]. The diabetic group in this experiment had notably elevated levels of hepatic *CHOP* and *caspase-9* expression. Administration of *D. aucheri* significantly reduced these levels. In addition, the expression of BCL2, which is recognized for its ability to prevent cell death, was reduced after the onset of diabetes. The decline was effectively counterbalanced by the therapy with *D. aucheri*. Conversely, *NF-κB* is crucial in the expression of inflammatory mediators. Furthermore, there have been reports of the interaction between the ERS and UPR pathways with the *NF-κB* [24]. Consistent with our predictions, our findings demonstrated an increase in *NF-κB* mRNA levels following the induction of diabetes in rats. Nevertheless, the administration of *D. aucheri* significantly reduced the elevated expression. The results suggest that the administration of *D. aucheri* extract with metformin modulates the gene expression of key components in ERS and apoptotic pathways, also simultaneously improving the UPR in the liver of diabetic rats.

Currently, there are few studies on the effects of the *D. aucheri* extract on the stereological liver tissue. Quantitative microscopic or stereological research provides useful insights into the effects of various medications on different tissues, while also preserving the cellular milieu, particularly in specialized tissues [63]. Compared to the control group, Figure 3 shows that liver stereological parameters such as liver weight, liver volume, hepatocyte volume, central vein volume, and sinusoid volume decreased in the diabetes group. Additionally, liver injury parameters, including tissue inflammatory volume and necrotic and fibrotic tissue volume, showed an increase in the liver tissues. These results are consistent with previous studies [64, 65].

The results of our study showed that there was no statistically significant variation in the volumetric characteristics of the liver structural components between the diabetic rats treated with *D. aucheri* and the control group. The study conducted by Ahmed OM et al. found that the impact of hydroethanolic extracts from both the leaf and flower head of *Cynara scolymus* on diabetic rats treated with nicotinamide/STZ was comparable [65].

A histological investigation conducted on rats with diabetes revealed a rise in liver damage markers. Several indicators observed in this study include fibrosis, necrosis, enlargement of sinusoidal gaps (shown by the thin arrow), accumulation of blood cells, and a higher presence of inflammatory cells compared to the healthy control group. The findings were consistent with the results of Mahata et al. [66]. The administration of *D. aucheri* and metformin successfully restored these alterations. However, the improvement was more pronounced with a dosage of 500 mg/kg as compared to 250 mg/kg. These data indicate that the extract, as well as the hydroethanolic extracts of *Cynara scolymus* leaf and flower head, is concluded to have similar effects [65]. The treatment of *D. aucheri* can greatly influence the stereological changes in the structural components of the liver tissue induced by diabetic rats. Therefore, *D. aucheri* reverses the alterations back to their original state, presumably indicating its defensive impact against tissue damage caused by diabetes. The study should accept certain limitations while emphasizing the possible benefits of *D. aucheri* on liver damage, specifically concerning ER stress, UPR, and apoptotic pathways. The study employed qRT‐PCR to determine the level of mRNA expression of cell signaling markers. Western blot analysis can be employed to further confirm the reported findings. In addition, accurately measuring the protein composition in each signaling pathway offers a comprehensive overview and aids in confirming the results. Moreover, the use of a crude hydroalcoholic extract without advanced phytochemical characterization (e.g., HPLC or LC‐MS analysis) should also be considered a limitation of the present study.

## 5. Conclusion

The findings of our investigation demonstrate that the hydroalcoholic extract of *D. aucheri* reduces blood glucose levels and improves liver enzymes and lipid profile in diabetic rats. In addition, it attenuates inflammatory markers, suggesting an improvement in the hepatic inflammatory state. These protective effects may be associated with the modulation of UPR/ER stress‐related pathways; however, further confirmation at the protein level is required to support the mechanistic interpretation. Overall, the results indicate a beneficial effect of the extract in this experimental diabetic model.

**Nomenclature**
ERS: Endoplasmic reticulum stress
UPR: Unfolded protein response
PERK: Protein kinase RNA‐like ER kinase
IRE1: Inositol‐requiring enzyme type 1
XBP1: X‐box–binding protein 1
*GRP78*
: 78‐kDa glucose‐regulated protein
ATF6: Activating transcription factor 6
*CHOP*
: CCAAT/enhancer‐binding protein homologous protein
BCL2: B‐cell lymphoma 2
BCL‐XL: B‐cell lymphoma extra‐large
MCL‐1: Myeloid cell leukemia sequence 1
BIM: BCL2‐interacting mediator of cell death
ATF4: Transcription factor 4
AP‐1: Activator protein‐1
STZ: Streptozotocin
i.p: Intraperitoneally
ALT: Alanine aminotransferase
AST: Aspartate transaminase
ALP: Alkaline phosphatase
FBS: Fasting blood sugar
TG: Triglycerides
MDA: Malondialdehyde
TAC: Total antioxidant capacity
SOD: Superoxide dismutase
CAT: Catalase
GPX: Glutathione peroxidase
HDL: High‐density lipoprotein
LDL: Low‐density lipoprotein
Chol: Cholesterol
IL‐6: Interleukin‐6
IL‐1β: Interleukin 1 beta
TNF‐ α: Tumor necrosis factor alpha
*NF-κB*
: Nuclear factor kappa‐light‐chain‐enhancer of activated B cells
qRT‐PCR: Quantitative real‐time polymerase chain reaction

## Author Contributions

All experiments, statistical analysis, and figure preparation were conducted by Alireza Raeisi, Farhad Koohpeyma, Amir Maleksabet, and Sanaz Dastghaib. The initial draft of the manuscript was written by Roozbeh Kiani, Somayeh Igder, Narges Rezaei, and Nima Rahimikashkooli. All tests were set up by Alireza Raeisi and Sanaz Dastghaib, and second draft of manuscript was written by Somayeh Igder, and Sanaz Dastghaib. A final manuscript proof was completed by Mesbah Shams and Sanaz Dastghaib.

## Funding

This work was financially supported by the Shiraz University of Medical Sciences (Grant No. 25758).

## Disclosure

All authors have read and agreed to the published version of the manuscript.

## Consent

The authors have nothing to report.

## Conflicts of Interest

The authors declare no conflicts of interest.

## Data Availability

This article contains all the data created and examined throughout this investigation. The corresponding author will provide the datasets used or analyzed during the current work upon reasonable request.
